# From Hawaii to PECASE award: tips of success from a female bioinformatician

**DOI:** 10.1186/s13059-019-1886-x

**Published:** 2019-12-12

**Authors:** Lana X. Garmire

**Affiliations:** 1 Previous address: University of Hawaii Cancer Center, 701 Ilalo Street, Honolulu, 96801 USA; 2 Present address: Department of Computational Medicine and Bioinformatics, University of Michigan, Ann Arbor, MI 48105 USA

Females are much under-represented in computational science fields, including Bioinformatics. Despite the promotion of gender equality in faculty hiring, women are faced with unique sets of challenges throughout the career development pipeline, such as lack of the support system in child-raising and lack of mentorship and advocate (esp. from females) for their career success. Over the course of my tenure-track faculty positions, I have had several points of reflection on the journey that I took as a female faculty in a male-dominated field and as a working mom in a very demanding profession. In writing up my experience thus far, I hope to encourage junior female scientists to continue down this path.

There were numerous times in my early career where I felt that I would not make it, but through perseverance I found a way through. When I started my graduate school in UC-Berkeley in 2001, I worked very hard in a famous breast cancer experimental lab, but I was hopelessly lost: experiments never seemed to work however hard I tried. I then recalled that my math teacher had told me to never give up mathematics (as I had aced my advanced math class), and I decided to give Bioinformatics, a very new field, a try. This move was very bold, because I had grown up in China without much experience with computers. I had to take many classes in statistics, mathematics, and computer science in order to make the biggest career transition in my life and earn my PhD. During my postdoc at UC-San Diego, I had the notorious “two-body” (or dual-career) problem, as my husband started his tenure-track position in the University of Hawaii. This meant that I had to take on the almost impossible task of finding myself a faculty position in Hawaii, where there is only one R1 research university. After taking a detour in industry for a year, in 2012, I got an on-site interview and was offered a tenure-track position in the University of Hawaii Cancer Center! The following years (2012 to 2017) were racing for tenure while raising a young kid, in the most beautiful yet remote state of the USA. Today, it feels surreal that all this hard work has paid off and I was awarded the prestigious Presidential Early Career Award for Scientists and Engineers in 2019. In the following sections, I will share my five tips of success that are particularly important for female scientists given their unique challenges. For more general career advice, please refer to earlier publications [1, 2]:

1. *You can have a career and a family at the same time.* Despite the efforts in gender equality movements, the stereotypes are still prevailing that females need to choose between being wives and professional/faculty, or between being mothers and professional/faculty. During my postdoc and the postpartum periods, I was given voluntary advice to give up the idea of working or becoming a faculty and become a housewife, by males and females, relatives or non-relatives, those with or without PhDs. You *have to* be true to yourself, be courageous, and ask what will really make *you* happy, regardless what others say, even though you may feel discouraged. It is challenging to balance between work and family life, but you can certainly do it! Many female faculties have walked this path, and you are not alone.
  In order to succeed in this journey, you will need a supportive team. Do not be afraid to ask for help from your partner, your family and extended family, your employer, and the social network. You should do thorough homework and plan well ahead. You will need to look for a daycare or a trusting nanny, probably as soon as you know you will be a parent. With your infant, you will need to make adjustments to attend conferences or go to NIH study sections. It has been good to see a changing climate at conferences where services are offered for parents of young children, such as on-site babysitting or family viewing rooms. However, there will still be times when you will need to make the arrangements to hire a babysitter at the hotel when you attend the meeting. It is also possible to attend NIH study sections remotely.
  The reality is that you cannot fulfill all your roles all at the same time, and it is OK to not be perfect. You should focus on your priorities, such as yourself, your family, and work, and you should not feel guilty for paying for services such as house cleaning, meal preparation, or babysitters. Overall, being a parent made me a more efficient and better researcher, as I became more focused at work. By better prioritizing my work, I grew to be a better parent for my children, which I find just as rewarding.
2. *Turn “two-body problems” into two-body teamwork.* Many of the female faculty I know have “two-body problems.” When they look for faculty positions, their partners also need to find academic or industrial positions in the vicinity. This is probably the toughest personal situation for anyone, and there is not a simple universal solution. You and your partner need to work together as a team to find what works the best for you together. It is a good strategy for the more senior one between you two to initiate the job search while the other applies for nearby positions. If you are research collaborators, it is a good idea to ask for joint hiring by presenting an integrated team plan. There may need to be some level of personal sacrifice, as one person may take the role as the main career driver and the other one may take a transition position. Please remember that this situation is not your fault, and if the potential employer is not willing to help, then you are probably better off getting a position elsewhere.
3. *Work efficiently and be disciplined.* It is needless to say that we must work hard to achieve tenure-track positions. I would like to stress here on “working efficiently”: with parenting obligations, your time to work is limited and you will need some strategies. What I found useful is to have long-term and short-term plans, set priorities properly, and self-reflect on them often. I start my weekly schedule regularly and commit to it, unless my kid(s) get sick or have to travel. I write down my daily to-do list and cross them out one by one, which gives much gratification. I minimize unnecessary meetings where I do not see my role in play quickly, and I prefer video conference calls rather than commuting whenever possible. As a parent of young kids, I do not have the luxury to travel often, so I target the high-quality, most relevant and small meetings, where I can really interact with researchers and establish relationships (such as the Pacific Biocomputing Symposium).
  One of the most influential writers of modern China said: “Time is like the water in a sponge; as long as you are willing to squeeze it, it always will have some (to come out)”. You should take advantage of the spare time between your child-care duties. For example, you may review a paper when you are waiting for your child outside their enrichment class, or work on an unfinished manuscript during the time your kids nap, or simply think about project ideas when you are patting your kids to sleep. I run around my kid(s) on the weekends, but I am much more efficient now than I was during my pre-child time.
4. *Be your own public relationship (PR) person.* Females are usually perceived as being conservative compared to male peers, especially in fields where they are so outnumbered. We need to act proactively to change this perception by communicating and self-advertising more, in order to increase our scholarly visibility. The conventional approaches are going to conferences or giving invited seminars to institutes. But there are newer avenues, such as social media. Tools like Linkedin, Twitter, blogging, and Youtube videos, and online communities like slack groups, to name a few, are good alternative approaches to share scientific updates, and you do not have to travel to meetings to promote your work.
  I am a big fan of open science, and a lot of our work is disseminated in the form of preprints before getting published. Once the preprints are available, I tweet it online with URL links and figure snapshots, to engage the research community. You can also use a more formal PR channel such as LinkedIn, which lets users update what is new in their statuses. It is important to have a regularly updated research website that shows visitors any news from your research group, such as publications, awards, and grants. These online tools have really helped to make my work known even though I was living in Hawaii, 2000 miles away from the closest continent. It also helped to keep up with friends, get in touch with new collaborators, attract trainees, and be updated with the newest developments in the field.
5. *Challenge and improve yourself.* One of the best pieces of advice I got from my faculty mentor was “Do not settle”. The field of Bioinformatics is constantly evolving, closely following after the development of genomics technologies. As a PI, you have to train yourself to be open-minded and willing to learn constantly, both scientifically and administratively. For example, you should actively reach out to more senior (likely male) peers for collaborations. There is nothing to lose to reach out, and often people are willing to help. At conferences or seminars, do not feel shy to ask questions, as we are all there to learn. For the projects, rather than asking yourself what you feel comfortable to do, ask instead what is needed to get the scientific questions answered, and engage all the resources to answer those questions. Since turning myself into a computational researcher, I had not thought that one day, I would run a wet lab, but now I do because we need that component to generate data for our projects. Lastly, I would like to share from my own experience that you should not compare yourself with others, and instead compare yourself from yesterday to today. As long as you keep improving yourself, you will be successful in your own way.
